# Prevalence of Intimate Partner Violence in Pregnancy: An Umbrella Review

**DOI:** 10.3390/ijerph18020707

**Published:** 2021-01-15

**Authors:** Rosario M. Román-Gálvez, Sandra Martín-Peláez, Juan Miguel Martínez-Galiano, Khalid Saeed Khan, Aurora Bueno-Cavanillas

**Affiliations:** 1 Departamento de Enfermería, Facultad de Ciencias de la Salud, Universidad de Granada, 18071 Granada, Spain; galvezmaro@gmail.com; 2 Unidad Asistencial Churriana de la Vega, Servicio Andaluz de Salud, Churriana de la Vega, 18194 Granada, Spain; 3 Departamento de Medicina Preventiva y Salud Pública, Facultad de Medicina, Universidad de Granada, 18071 Granada, Spain; profkkhan@ugr.es (K.S.K.); abueno@ugr.es (A.B.-C.); 4 Instituto de Investigación Biosanitaria de Granada IBS, 18014 Granada, Spain; 5 Department of Nursing, University of Jaén, 23071 Jaén, Spain; jgaliano@ujaen.es; 6 Consortium for Biomedical Research in Epidemiology and Public Health (CIBERESP), 28029 Madrid, Spain

**Keywords:** intimate partner violence, pregnancy, prevalence, umbrella review

## Abstract

Background: Intimate partner violence (IPV) is a public health concern, especially during pregnancy, and needs to be urgently addressed. In order to establish effective actions for the prevention of IPV during pregnancy, authorities must be aware of the real burden of IPV. This review aimed to summarize the existing evidence about IPV prevalence during pregnancy worldwide. Methods: A review of reviews was carried out. All published systematic reviews and meta-analyses published until October 2020 were identified through PubMed, Scopus, and Web of Science. The main outcome was the IPV prevalence during pregnancy. Results: A total of 12 systematic reviews were included in the review, 5 of them including meta-analysis. The quality of the reviews was variable. Physical IPV during pregnancy showed a wide range (1.6–78%), as did psychological IPV (1.8–67.4%). Conclusions: Available data about IPV prevalence during pregnancy were of low quality and showed high figures for physical and psychological IPV. The existing evidence syntheses do not capture the totality of the worldwide disease burden of IPV in pregnancy.

## 1. Introduction

Intimate partner violence (IPV), defined as physical violence, sexual violence, harassment, and psychological assault (including coercive tactics) by a current or former intimate partner [1], is a public health concern that urgently needs to be addressed. During pregnancy, the woman experiences a situation of special dependence, both physical and emotional. In this period, exposure to violence affects not only the mother but also the fetus, which is at greater risk than in other stages of life [2]. In fact, IPV has been associated with adverse pregnancy outcomes including increased risk of human immunodeficiency virus infection [3], perinatal depression [4], insufficient weight gain during pregnancy [5], uterine rupture, hemorrhage, maternal death [6], prematurity, low birth weight, newborns small for gestational age [7], stillbirth [8], and reduced levels of breastfeeding [9]. At the same time, routine contacts with the health system offer an excellent detection window to identify it and establish protective measures. Despite this, IPV during pregnancy is a neglected condition, even though it is more common than many maternal health conditions like preeclampsia and gestational diabetes [10].

IPV during pregnancy should be an avoidable global public health problem. However, in order to establish effective actions for the prevention of IPV during pregnancy, such as the performance of systematic screening and diagnosis of IPV in the antenatal visits, authorities must be aware of the real worldwide burden of IPV. However, information about IPV prevalence is not consistent. Whereas some studies indicate higher prevalence of IPV during pregnancy than before [11] or after [12,13] the pregnancy, other studies report a smaller prevalence [14,15]. Furthermore, the prevalence of IPV during pregnancy is reported to vary depending on the definition used [1], the screening strategy [16,17], and the development status of the population studied [10,18]. These factors make comparison between individually reported rates difficult.

This review aims to summarize the existing evidence about IPV prevalence during pregnancy worldwide through a synthesis of systematic reviews and meta-analysis. Prevalence studies provide a snapshot of a situation in a specific context, so it is important to bring together different existing studies for a global understanding. This work analyzes the existing reviews, identifying their strengths and limitations and laying the foundations for future reviews that clarify the situation of IPV during pregnancy in a complete and realistic way.

## 2. Materials and Methods 

This umbrella review was written according to the Preferred Reporting Items for Systematic Reviews and Meta-Analyses (PRISMA) statement for systematic reviews and meta-analyses [19] and the Aromataris’ guidelines for performing umbrella reviews [20]. 

### 2.1. Inclusion and Exclusion Criteria 

Systematic reviews and meta-analyses including observational studies reporting IVP prevalence suffered by women during pregnancy were considered in this review. The types of IPV were classified as physical, sexual, psychological and any type of IPV. Studies not following a systematic review approach, narrative reviews, and primary studies were excluded. No language restrictions were applied in this review.

### 2.2. Literature Search and Selection of Studies

Relevant systematic reviews and meta-analyses according to inclusion criteria were identified through systematic searches of the following electronic databases: PubMed, Web of Science and Scopus, CYNAHL, PsycINFO, Social Science Database, and Sociological Abstracts. The full search strings can be found in Table 1. When the search engine used only allowed selecting systematic reviews or meta-analyses, the terms “systematic reviews” OR “meta-analyses” were not included in the search string; otherwise, those terms were included. Studies published from inception until October 2020 were included. Reference lists of identified studies were checked. 

### 2.3. Data Collection and Analysis

Eligible studies were selected through a multistep approach (elimination of duplicates, title reading, abstract and full-text assessment). Two researchers (S.M.-P. and R.M.R.-G.) independently examined titles and abstracts, evaluating afterwards full texts according to the inclusion criteria described above. Any disagreement between the reviewers was resolved by means of a consensus session with a third reviewer (A.B.-C.). In case of ambiguity in reporting or lack of data, primary authors were contacted for clarification.

### 2.4. Data Extraction and Management

Data were independently extracted by two researchers (S.M.-P. and R.M.R.-G.), and the following information was considered for each article: (1) first author and year of publication; (2) interval of time covered by the review; (3) countries (of studies included in the systematic review or meta-analysis); (4) number of studies included; (5) study design (of studies included in the systematic review or meta-analysis); (6) sample characteristics; (7) IPV as main outcome; (8) type of IPV investigated; (9) meta-analysis performance; (10) IPV outcome.

### 2.5. Quality Assessment Tools

The updated AMSTAR 2 version for systematic reviews and meta-analyses was used to evaluate the methodological quality and risk of bias of studies included in the systematic review [21]. The overall final rating of each systematic review was judged as high, moderate, low, or critically low. In case of disagreements, a consensus session with the third reviewer (A.B.-C.) was held.

## 3. Results

**Figure 1:**
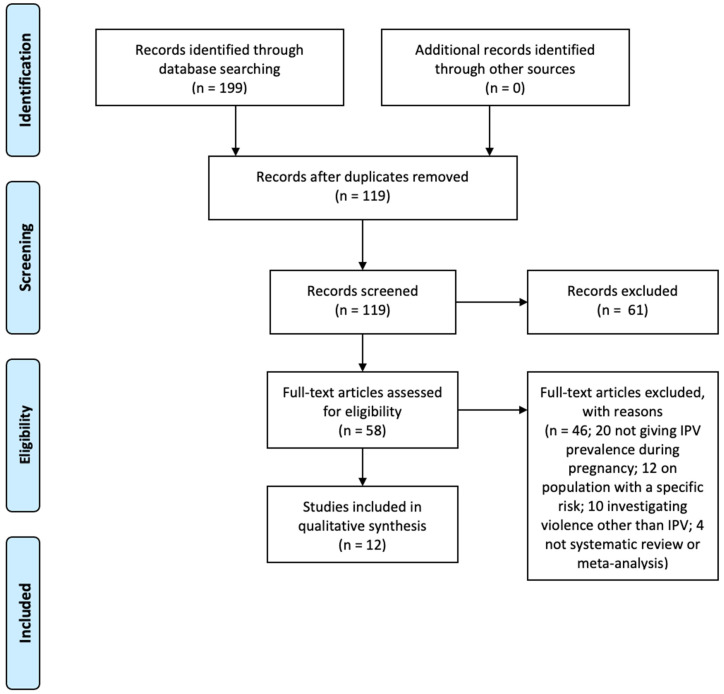
PRISMA flowchart of the study selection process.

The electronic search initially resulted in 199 citations. A total of 80 studies were excluded after elimination of duplicates. From the 119 remaining, 61 were excluded after title and abstract screening and 58 full-text articles were selected and read. From those, a total of 12 systematic reviews were included in this umbrella review, of which 5 were meta-analyses. The reasons for exclusion were the lack of data about IPV prevalence during pregnancy (20), the use of violence other than IPV or the indistinct report of IPV or domestic violence (10), investigations on populations with a specific risk, supposedly different from the general population (12), and not being a systematic review or meta-analysis (4). The list of the excluded articles is presented in Supplementary Materials Table S1. Figure 1 shows the PRISMA flowchart and the study selection process. 

Characteristics of included systematic reviews and meta-analyses are shown in Table 2. Only two reviews included global data [22,23], most of which were limited to a country or a group of countries, mainly from Asia [4,23,24,25,26,27,28] and Africa [4,26,27,29,30], followed by America [31,32], Europe [32], and Australia [32]. The number of studies included in the reviews giving information about IPV prevalence during pregnancy ranged from 2 [24] to 73 [23]. 

Most of the studies included in the selected reviews were cross-over studies, in which there was only a single evaluation of the women sometime during pregnancy. Some of the reviews also included cohort studies [4,24,25,30,31,32]. Reviews included studies giving information about IPV only at pregnancy [22,23,24,27,29,30], both during pregnancy or at postpartum [4,30,31,32], during pregnancy, or having a child 2 years old or younger [25] or at current pregnancy or any pregnancy [26].

Wide differences were also observed regarding the type of IPV violence investigated. Of the selected reviews, nine investigated prevalence of physical violence [4,22,24,25,26,27,28,30,31], nine psychological violence [4,22,24,25,26,27,28,30,31], ten sexual violence [4,22,23,24,25,26,27,28,30,31], and three any type of violence [26,29,32]. From the selected studies, four did not report IPV pregnancy during pregnancy as main outcome [24,25,26,32]. Five studies showed a summarized estimate of IPV during pregnancy [23,24,28,29,30]. In most of the reviews ranges of IPV prevalence are given [4,22,24,26,27,31,32].

From the reviews included in the study, many showed data about any IPV prevalence worldwide. The data about prevalence of any kind of IPV during pregnancy were obtained from different countries [26,29,32]. The highest range of any IPV prevalence was obtained in Portugal, USA, and Australia [32] (15.4–40%), followed by Ethiopia [29] (26.1% (95% CI: 20–32.3)) and countries from the Arab League [26] (40.9–44.1%).

Regarding physical IPV during pregnancy, China [28] and Vietnam [25] showed the lowest ranges; (3.6% (95% CI: 1.6–6.2%)) and (3–8.5%) respectively. Higher ranges were found in countries from Latin America [31] (2.5–38.7%), Africa and Asia [4] (2–35%) followed by low- and middle-income countries [27] (5–52.8%), countries from the Arab League [26] (10.4–34.6%) and African countries [30] (22.5–40%), being the widest range the one found in Saudi Arabia [24] (21–78%). James and colleagues [22] showed a prevalence of 13.8% in all over the world.

As for any and physical IPV, China [28] showed the lowest and smallest ranges of psychological IPV prevalence during pregnancy (4.2% (95% CI: 1.8–7.5%)). Higher ranges were found in Vietnam [25] (6–32.5%) and countries from the Arab League [26] (23.4–32.6%), Latin America [31] (13–44%), and African countries [30] (24.8–49%). The widest ranges were found in low- and middle-income countries [27] (17–67.4%) and countries from Asia and Africa [4] (22–65%). James and colleagues [22] showed a prevalence of 28.4% throughout the world.

In general, sexual violence showed lower ranges of prevalence than the other types of IPV violence during pregnancy, being the lowest in China [28] (1.3% (95% CI: 0.6–2.5%)), followed by Vietnam [25] (3.4–10%), countries from the Arab League [26] (5.7–15.0%), low- and middle-income countries [27] (2.8–21%), Africa [30] (2.7–26.5%) and Latin America [31] (3–34.4%). The highest and widest ranges of sexual IPV prevalence during pregnancy were found in countries from Asia and Africa in the study of Halim and colleagues [4] (9–40%). Worldwide, prevalence of sexual IPV during pregnancy remained lower than 18% [22,23].

Quality assessment is reported in Table 3. Although all of the studies used a comprehensive literature search strategy, only two of the selected reviews did not include the components of PICO in their research questions and inclusion criteria [22,24], the reviews described the included studies in adequate detail, with the exception of three studies [22,26,32], and in only three of the reviews [22,25,27], authors did not report any statement about potential sources of conflict of interest. For some other aspects of the AMSTAR2 checklist, the quality remained low. Thus, none of the reviews included an explicit statement that the review methods were established prior to the conduct of the review nor the sources of funding for the studies included. Only one review provided a list of excluded studies and justified the exclusions [28]. Only three of the reviews explained their selection of the study design for inclusion in the review [24,25,26]. Half of the reviews performed study selection in duplicate [4,23,24,25,26,28], whereas only three did not perform data extraction in duplicate [4,22,31]. Half of the studies included did not use a satisfactory technique for assessing the risk of bias in individuals included in the review [22,23,27,29,31,32], and more than half did not account for risk of bias in individual studies when interpreting/discussing the results of the review [22,23,24,25,31,32,33]. Only two of the reviews provided a satisfactory explanation for, and discussion of, any heterogeneity observed in the results of the review [22,26]. In the reviews where meta-analysis was performed, the authors used appropriate methods for statistical combination of results [23,25,28,29,30], but in only three of them, the review authors assessed the potential impact of risk of bias in individual studies [25,28,30] and only two [28,29] carried out an adequate investigation of publication bias.

## 4. Discussion

The aim of this umbrella review was to provide a summary of the evidence currently available on global IPV prevalence in women during pregnancy. Despite the fact that the selected reviews were recent, they are of low quality as assessed against most of the AMSTAR2 recommendations. There were only two reviews giving worldwide IPV prevalence during pregnancy [22,23], both of them complying with less than half of the AMSTAR2 criteria.

### 4.1. Limitations

We selected systematic reviews for prevalence of IPV in pregnancy, yet we obtained very diverse data. In the reviews, sometimes the concepts of IPV and domestic violence were mixed together. Most of the included studies were cross-sectional self-report surveys, which may have been associated with inaccurate recall [24,28,31]. They did not always specify the gestational time point of IPV evaluation. It was common to find a mix among studies assessing IPV at any time of pregnancy or even after pregnancy. This is important since data can vary depending on the gestational age when IPV is measured, antenatally or after delivery. One review [4] also included studies that assessed violence for a period that is inclusive of, but not exclusive to, pregnancy.

Some of the included reviews, in spite of being systematic reviews, showed possible bias in studies included for evidence synthesis. They generally failed to adequately address the heterogeneity of results [22,28,32]. Others had a very narrow geographical coverage [24,25,26,29]. In addition, sample sizes of the included studies were generally small [29,30,31], and the use of standardized and validated IPV instruments was low. Geographical coverage of the reviews selected was mainly focused on low-income countries, a fact that invites readers to infer that IPV is a problem exclusive of those countries, which is far from the reality [34].

The main strength is that we have conducted an umbrella review following up the PRISMA and Aromataris’ guidelines. Our search has been exhaustive, collecting all kind of IPV.

### 4.2. Implications

Whereas the consequences of IPV during pregnancy on the mother and on the newborn are widely known [3,4,5,6,7,8,9], the frequency and types of IPV in that period are not fully characterized. WHO recommendations on antenatal care for a positive pregnancy experience advise considering clinical inquiry about the possibility of IPV at antenatal care visits when assessing conditions that may be caused or complicated by IPV [35]. Other prenatal care guidelines affirm that clinical practitioners should be aware of the possibility of IPV, but do not include any specific recommendation related to the screening [36]. It is well known that IPV is associated with adverse mental health and obstetrical health consequences for the mother, fetus, and child, but women are reluctant to speak about this topic without a previous inquiry [37]. The American College of Obstetricians and Gynecologists guidelines recommend screening for IPV at the first prenatal visit, at least once per trimester, and at the postpartum checkup [38]. However, the overall rate of screening asymptomatic women is distressing [39]. Due to the high prevalence of this serious problem, estimated violence during pregnancy ranges from 15 to 40.5% for any type of violence, figures higher than those previously reported by Perttu et al. [40]; it is vital to have a correct estimation of its magnitude. These evaluations are necessary to underscore the importance of systematic screening: only when health staff are aware of the right prevalence and repercussion of IPV will they be able to cope with the screening barriers and to identify the most vulnerable populations by introducing screening programs in antenatal care.

Isolated prevalence studies may underestimate the true IPV prevalence due to barriers to open disclosure. These barriers could vary in different cultures and religions; e.g., widespread social norms in some regions support husbands’ right to physically discipline wives. Abused women often face high social, economic, and legal barriers to divorce, a situation that is made worse by unresponsive law enforcement and health care institutions. In this social context, women are often reluctant to report violence to authorities and may hesitate to disclose violence to survey interviewers. In many societies, women are also reluctant to report violence because they are ashamed of living under this kind of relationship.

Summarizing the figures of IPV prevalence in pregnancy is needed to highlight the public health importance of this problem, with rates in some studies reported to be over 50%. These figures point towards the need of systematic screening in pregnancy. However, the analysis of published IPV reviews showed weaknesses in the research available on this topic. This umbrella review allows us to identify some methodological aspects that should be addressed in future reviews, related to geographic scope, study selection, and bias assessment.

## 5. Conclusions

Available data about IPV prevalence during pregnancy are of low quality. The existing evidence syntheses do not capture the totality of the disease burden in IPV in pregnancy. Despite there being wide variability in existing prevalence figures, it is worth noting that no less than 1 out of 50, and as many as 1 out of 2 women, could be suffering physical IPV in pregnancy. Psychological IPV violence is reported to be even more frequent in the published reviews. The existing evidence syntheses do not capture the totality of the worldwide disease burden of IPV in pregnancy.

## Figures and Tables

**Table 1:** Search strings.

Database	Searching String
PUBMED	(“Intimate Partner Violence” [Mesh]) AND ((“Pregnancy” [Mesh]) OR (“Pregnant Women” [Mesh]) OR (“Prenatal Care” [Mesh])) AND (“Prevalence” [Mesh])
Rest of databases	(“Intimate Partner Violence”) AND ((“Pregnancy”) OR (“Pregnant Women”) OR (“Prenatal Care”)) AND (“Prevalence”)

**Table 2:** Characteristics of the studies selected for the umbrella review of reviews of worldwide prevalence of IPV in pregnancy.

First Author, Year	Interval of Time	Country	Studies Included (n)	Study Design Included	Population Characteristics	IPV in Pregnancy Main Outcome Y/N	Type of Violence	Meta-Analysis Y/N	IPV Outcome
Alebel et al., 2018 [29]	Inception–February 2018	Ethiopia	8	Observational	Pregnant women	Y	Any	Y	26.1% (95% CI: 20, 32.3)
Alhalal et al., 2019 [24]	Inceptio–March 2018	Saudi Arabia	2 *	Cross-sectional Case-control Cohort	Pregnant women	N	Ph, Ps, S	N	Ph, 21–78% (other kind of violence figures provides only by an author)
Bazyar et al., 2018 [23]	1997–2015	World and Iran	73	Any	Pregnant women	Y	S	Y	World 17% (95% CI:15–18%) Iran 28% (95% CI: 23–32%).
Do et al., 2019 [25]	1970–2018	Vietnam	8	Cross-Sectional Cohort	Pregnant women or having a 2-year or younger child	N	Ph, Ps, S	Y	Ph, 3–8.5% Ps, 6–32.5% S, 3.4–10%
Elghossain, 2019 [26]	January 2000–January 2016	Arab league countries	7 *	Facility-based studies	Current pregnancy; Any pregnancy/any perpretator	N	Ph, Ps, S, Any	N	Ph, 10.4–34.6% Ps, 23.4–32.6% S, 5.7–15.0% Any, 40.9–44.1%
Han and Stewart, 2014 [31]	Inception–March 2012	Peru; Bolivia; Colombia; Brazil; Mexico; Guatemala; Costa Rica; Nicaragua; Haiti; and Dominican Republic.	31	Cross-sectional Cohort Case-control	Women during pregnancy or at postpartum	Y	Ph, Ps, S	N	Ph, 2.5–38.7% Ps, 13–44% S, 3–34.4%
Halim et al., 2018 [4]	February 1990–May 2007	Bangladesh, India, Nepal, Pakistan, Vietnam, Tanzania, Ethiopia, Malawi, Zimbabwe and Egypt	24	Cross-sectional Cohort	Women during pregnancy or at postpartum	Y	Ph, Ps, S	N	Ph, 2–35% Ps, 22–65% S, 9–40%
James et al., 2013 [22]	NR	All over the world		NR	Pregnant women	Y	Ph, Ps, S	N	Ph, 13.8% Ps, 28.4% S, 8.0%
Méndez-Figueroa et al., 2013 [32]	January 1990–May 2012	Australia Portugal USA	5	Case-control Cohort	Women during pregnancy and postpartum	N	Any	N	15.4–40%
Shamu et al., 2011 [30]	2000–2010	Nigeria, South Africa, Zimbabwe, Uganda, Rwanda	19	Cross-section Cohort Case-control RCT	Pregnant women or within two months of giving birth	Y	Ph, Ps, S	Y	Ph, 22.5–40%. Ps, 24.8–49% S, 2.7–26.5%
Udmuangpia et al., 2020 [27]	January 2009–May 2018	Bangladesh, Colombia, Ethiopia, Kenya, India, Iran, Nepal, Rwanda, and Uganda	12	Cross-sectional Qualitative studies	Pregnant women 15–24 year old	Y	Ph, Ps, S	N	Ph, 5–52.8% Ps, 17–67.4% S, 2.8–21%
Wang et al., 2017 [28]	Inception–January 2016	China	12	Cross-sectional	Pregnant women	Y	Ph, Ps, S	Y	Overall, 7.7% (95% CI: 5.6–10.1%) Ph, 3.6% (95% CI: 1.6–6.2%) Ps, 4.2% (95% CI: 1.8–7.5%) S, 1.3% (95% CI: 0.6–2.5%)

NR, not reported; Ph, Physical violence; Ps, Psychological violence; S, Sexual violence. * Number of studies included in the review related to IPV during pregnancy, when in the review reports IPV during pregnancy and others.

**Table 3:** Evaluation of selected IPV during pregnancy reviews based on AMSTAR 2 guidelines.

	AMSTAR 2 Checklist Items *															
Author, Year	1	2	3	4	5	6	7	8	9	10	11	12	13	14	15	16
Alebel et al., 2018 [29]	Y	N	N	Y	N	Y	N	Y	N	N	Y	N	Y	N	Y	Y
Alhalal et al., 2019 [24]	N	N	Y	Y	Y	Y	N	Y	Y	N	NA	NA	N	N	NA	Y
Bazyar et al., 2018 [23]	Y	N	N	Y	Y	Y	N	Y	N	N	Y	N	N	N	N	Y
Do et al., 2019 [25]	Y	N	Y	Y	Y	Y	N	Y	Y	N	Y	Y	N	N	N	N
Elghossain, 2019 [26]	Y	N	Y	Y	Y	Y	N	N	Y	N	NA	NA	Y	Y	NA	Y
Han and Stewart, 2014 [31]	Y	N	N	Y	N	N	N	Y	N	N	NA	NA	N	N	NA	Y
Halim et al., 2018 [4]	Y	N	N	Y	Y	N	N	Y	Y	N	NA	NA	Y	N	NA	Y
James et al., 2013 [22]	N	N	N	Y	N	N	N	N	N	N	NA	NA	N	Y	NA	N
Méndez-Figueroa et al., 2013 [32]	Y	N	N	Y	N	Y	N	N	N	N	NA	NA	N	N	NA	Y
Shamu et al., 2011 [30]	Y	N	N	Y	N	Y	N	Y	Y	N	Y	Y	N	N	N	Y
Udmuangpia et al., 2020 [27]	Y	N	N	Y	N	Y	N	Y	N	N	NA	NA	Y	N	NA	N
Wang et al., 2017 [28]	Y	N	N	Y	Y	Y	Y	Y	Y	N	Y	Y	Y	N	Y	Y

* Each number corresponds with an AMSTAR 2 checklist item as follows: 1. Did the research questions and inclusion criteria for the review include the components of PICO? 2. Did the report of the review contain an explicit statement that the review methods were established prior to the conduct of the review and did the report justify any significant deviations from the protocol? 3. Did the review authors explain their selection of the study designs for inclusion in the review? 4. Did the review authors use a comprehensive literature search strategy? 5. Did the review authors perform study selection in duplicate? 6. Did the review authors perform data extraction in duplicate? 7. Did the review authors provide a list of excluded studies and justify the exclusions? 8. Did the review authors describe the included studies in adequate detail? 9. Did the review authors use a satisfactory technique for assessing the risk of bias (RoB) in individual studies that were included in the review? 9. Did the review authors report on the sources of funding for the studies included in the review? 10. If meta-analysis was performed did the review authors use appropriate methods for statistical combination of results? 11. If meta-analysis was performed, did the review authors assess the potential impact of RoB in individual studies on the results of the meta-analysis or other evidence synthesis? 12. Did the review authors account for RoB in individual studies when interpreting/discussing the results of the review? 13. Did the review authors provide a satisfactory explanation for, and discussion of, any heterogeneity observed in the results of the review? 14. If they performed quantitative synthesis did the review authors carry out an adequate investigation of publication bias (small study bias) and discuss its likely impact on the results of the review? 15. Did the review authors report any potential sources of conflict of interest, including any funding they received for conducting the review? NA, Not applicable.

## Supplementary Materials

The following are available online at https://www.mdpi.com/1660-4601/18/2/707/s1, Table S1: List of excluded articles.
