# Supplementary material for: Prevalence of Intimate Partner Violence in Pregnancy: An Umbrella Review
Source: Int J Environ Res Public Health. 2021 Jan 15;18(2):707. doi: 10.3390/ijerph18020707 (PMC7830915; doi:10.3390/ijerph18020707)
Supplement: Supplementary file 1 [file ijerph-18-00707-s001.pdf]

Table S1. List of excluded articles after reading of full texts.

	**Authors, year**	**DOI**	Reason for exclussion*
1	Alvarez-Segura et al., 2014	DOI 10.1007/s00737-014-0440-9	A
2	Anderson et al., 2017	DOI 10.1007/s00737-017-0723-z	B
3	Aztlan-James et al., 2017	doi:10.1016/j.whi.2017.02.002.	A
4	Bayrampour et al., 2018	https://doi.org/10.1080/02646838.2018.1492097	A
5	Bundock et al., 2020	DOI: 10.1177/1524838018770412	B
6	Dadi et al., 2020	https://doi.org/10.1371/journal. pone.0231940	A
7	Dadi et al., 2020	https://doi. org/10.1371/journal.pone.0227323	A
8	Donovan et al., 2016	DOI: 10.1111/1471-0528.13928	A
9	Fisher et al., 2012	doi:10.2471/BLT.11.091850	B
10	Geller and Stasko, 2017	http://dx.doi.org/10.1016/j.jogn.2017.04.136	D
11	Grace and Anderson, 2018	doi:10.1177/1524838016663935	A
12	Grace and Fleming, 2016	doi:10.1002/wmh3.209.	A
13	Gracia-Leiva et al., 2019	http://dx.doi.org/10.6018/analesps.35.2.333101	A
14	Hall et al., 2014	doi:10.1371/journal.pmed.1001581	B
15	Hawcroft et al., 2019	https://doi.org/10.1186/s12889-019-6619-2	C
16	Howard et al., 2013	doi:10.1371/journal.pmed.1001452	C
17	James-Hawkins et al., 2017	https://doi.org/10.1007/s00737-018-0843-0	A
18	Jha et al., 2018	https://doi.org/10.1016/j.ajp.2018.06.020	A
19	Jha et al., 2019	https://doi.org/10.1016/j.jacc.2019.01.041	B
20	Kassa et al., 2020	DOI: 10.1177/1524838018782205	B
21	Koirala and Chuemchit, 2020	http://doi.org/10.2147/IJWH.S235864	B
22	Kwok et al., 2016	http://doi.org/10.2147/IJWH.S235864	D
23	Mason-Jones et al., 2016	DOI: 10.1002/14651858.CD006417.pub3	B
24	Maxwell et al., 2015	DOI:10.1371/journal.pone.0118234	A
25	Meyer et al., 2019	http://dx.doi. org/10.1136/bmjopen-2018- 028809	D
26	Muluneh et al., 2019	doi:10.3390/ijerph17030903	C
27	Nia et al., 2017	http://jmums.mazums.ac.ir/article-1-10105-en.pdf	C
28	O'Doherty et al., 2015	DOI: 10.1002/14651858.CD007007	A

29	Orpin et al., 2020	DOI: 10.1177/1524838017731570	C
30	Pastor-Moreno et al., 2020	https://doi.org/10.1016/j.ajog.2019.07.045	B
31	Phares et al., 2029	https://doi.org/10.1016/j.amepre.2019.07.003	A
32	Popova et al., 2016	doi:10.1111/tmi.12755	A
33	Puente-Martínez et al., 2016	http://dx.doi.org/10.6018/analesps.32.1.189161	A
34	Quintanilla et al., 2016	http://dx.doi.org/ 10.1136/bmjopen-2016- 013270	B
35	Salam et al., 2016	http://dx.doi.org/10.1016/j.jadohealth.2016.05.022	A
36	Sapkota et al., 2017	DOI 10.1186/s13643-017-0657-6	C
37	Sapkota et al., 2019	https://doi.org/10.1186/s13643-019-0998-4	C
38	Semahegn and Mengistie, 2015	DOI 10.1186/s12978-015-0072-1	C
39	Semahegn et al., 2019	https://doi.org/10.1186/s12978-019-0726-5	C
40	Taft et al., 2012	http://www.biomedcentral.com/1471-2458/12/811	D
41	Takegata et al., 2017	doi:10.3390/healthcare5040091	B
42	Teixeira et al., 2019	http://dx.doi.org/10.1590/1806-9282.65.3.475	B
43	Tomás, 2016	DOI 10.1186/s12913-016-1423-5	A
44	Van Parys et al., 2014	doi: 10.1371/journal.pone.0085084	A
45	Wirtz et al., 2016	DOI 10.1186/s13031-016-0071-z	C
46	Yakubovich et al., 2018	doi:10.2105/ AJPH.2018.304428	A

*A, No data about IPV prevalence during pregnancy; B, Population with a specific risk supposedly different from the general population; C, Violence other than IPV or the indistinct report of IPV and domestic violence; D, Not systematic review or meta-analysis.
